# Effects of Gait Biofeedback Training on Spatiotemporal Gait Parameters in Stroke Survivors: A Systematic Review and Meta-Analysis of Randomized Controlled Trials

**DOI:** 10.3390/brainsci16070717

**Published:** 2026-07-03

**Authors:** Kaixiong Dai, Yuqiong Yang, Yujie Yang

**Affiliations:** School of Sports Medicine, Wuhan Sports University, Wuhan 430079, China; kaixiong127@163.com (K.D.); yuqiongyyu@163.com (Y.Y.)

**Keywords:** stroke, biofeedback, gait training, spatiotemporal gait parameters

## Abstract

**Highlights:**

**What are the main findings?**

- Gait biofeedback training may improve gait velocity in stroke survivors, with more tentative evidence for step length.
- The effect on gait cadence was unstable in sensitivity analysis, while no significant pooled effects were found for stride length or stance time.

**What are the implications of the main findings?**

- Current evidence suggests that gait biofeedback may be a promising adjunct to post-stroke gait rehabilitation, but the certainty of evidence varies across outcomes.
- Larger and better-reported trials are needed to clarify the effects on step length, cadence, stride length, and stance time, and to determine optimal feedback targets and training protocols.

**Abstract:**

**Background:** Stroke represents a major contributor to long-term disability and is commonly associated with impaired gait, balance, and mobility, which reduce independence and increase fall risk. Gait biofeedback training provides real-time performance-related feedback and may facilitate motor relearning. This study aimed to synthesize the available evidence of gait biofeedback training on spatiotemporal gait parameters in stroke survivors. **Methods:** PubMed, Embase, Web of Science, and the Cochrane Library were searched up to 7 April 2026. RCTs involving stroke survivors with gait impairment that compared gait biofeedback training with non-biofeedback rehabilitation and reported spatiotemporal gait outcomes were included. Risk of bias and certainty of evidence were assessed using RoB-1 and GRADE, respectively. Meta-analyses were conducted using mean difference (MD) with 95% confidence intervals (CIs). Heterogeneity was assessed using I^2^ and τ^2^, and 95% prediction intervals (PI) were calculated where possible. **Results:** 10 RCTs involving 304 participants were included. Compared with control interventions, gait biofeedback training may improve gait velocity (MD = 9.78 cm/s, 95% CI 6.06 to 13.50, *p* < 0.001, 95% PI 2.14 to 17.41) and step length (MD = 5.88 cm, 95% CI 1.14 to 10.61, *p* = 0.01, 95% PI −10.18 to 21.94). However, the certainty of evidence was stronger for gait velocity than for step length. A significant effect on cadence was observed in the primary analysis, but this finding was unstable in the sensitivity analysis. No significant pooled effects were found for stride length or stance time. The wide PI for step length, stride length, and stance time indicates that the expected effects may vary across future clinical settings. **Conclusions:** Gait biofeedback training may improve gait velocity after stroke. Evidence for step length improvement is more tentative, while evidence for cadence, stride length, and stance time remains insufficient or unstable. Additional well-designed high-quality RCTs are needed to confirm these findings and determine optimal feedback modes and training protocols. The review was registered in PROSPERO (CRD420261354683).

## 1. Introduction

Globally, stroke remains a major cause of mortality and sustained disability. Recent global estimates indicate that stroke continues to impose a substantial public health burden, with a growing number of survivors living with persistent functional limitations [1,2]. Among these sequelae, motor impairment, poor balance, and reduced walking ability are particularly prevalent and have major consequences for independence, community participation, and quality of life [3]. Consequently, restoration of safe and efficient walking is a central goal of post-stroke rehabilitation. In particular, post-stroke gait is commonly characterized by reduced gait velocity, shortened step and stride lengths, altered cadence, prolonged stance or stance time, and spatiotemporal asymmetry [4]. These gait abnormalities not only reduce locomotor efficiency but also increase fall risk and restrict social reintegration [5]. Accordingly, spatiotemporal gait parameters are widely recognized as clinically meaningful indicators for assessing gait impairment and rehabilitation-related changes after stroke [5,6].

Biofeedback gait training is an augmented-feedback intervention. In motor learning, augmented feedback is commonly divided into knowledge of results (KR) and knowledge of performance (KP) [7]. KR provides information about the outcome of a movement, such as walking speed or task success. In contrast, KP provides information about the quality or pattern of movement, such as limb loading, foot contact, trunk control, or gait symmetry. In post-stroke gait rehabilitation, biofeedback systems may provide KR, KP, or both. This distinction is important because gait biofeedback is often designed not only to increase walking speed, but also to help stroke survivors recognize and correct abnormal gait patterns during practice [8]. Depending on the training system, feedback may target spatiotemporal, kinematic, kinetic, or electromyographic variables and may be delivered through visual, auditory, or haptic feedback modalities, with haptic feedback being delivered as tactile or vibrotactile stimuli in real time. Compared with non-biofeedback rehabilitation, biofeedback-based interventions may offer several advantages, including greater task specificity, immediate knowledge of performance, enhanced patient engagement, and improved motor relearning through repeated error correction [9].

Functional endpoints such as walking endurance, balance, falls, and community ambulation are clinically important. However, these outcomes are influenced by many factors, including cardiovascular fitness, balance control, cognition, environmental demands, and daily activity demands. Therefore, they may not directly reflect the specific gait component targeted by biofeedback training. In contrast, spatiotemporal gait parameters, including gait velocity, step length, stride length, cadence, and stance time, are objective, commonly reported, and closely related to the feedback targets used in many gait biofeedback interventions. These parameters also provide a more detailed description of how walking changes after training. However, they should be interpreted as intermediate gait outcomes rather than complete measures of functional recovery. In particular, faster gait velocity does not necessarily mean that walking is safer, more symmetrical, or more energy-efficient.

Despite these potential advantages, the clinical effectiveness of gait biofeedback training after stroke remains incompletely understood. Existing trials differ considerably in feedback modality, target parameter, intervention dose, and control conditions, and outcome reporting is often inconsistent. Recent reviews suggest potential improvements in gait velocity, balance, and functional mobility following gait biofeedback. However, pooled evidence for specific spatiotemporal gait parameters remains limited and sometimes difficult to interpret [10,11].

Against this background, a focused systematic review and meta-analysis are warranted to clarify the effects of gait biofeedback training on key spatiotemporal gait parameters after stroke. By synthesizing parameter-specific evidence and examining the robustness of pooled estimates, the present study aims to provide a more clinically interpretable basis for the use of gait biofeedback training in stroke rehabilitation.

## 2. Methods

This review was prepared following the PRISMA (Preferred Reporting Items for Systematic Reviews and Meta-Analysis) 2020 statement and registered with the International Prospective Register of Systematic Reviews (PROSPERO) (Reference number: CRD420261354683; https://www.crd.york.ac.uk/PROSPERO/view/CRD420261354683 (accessed on 7 May 2026)).

### 2.1. Data Sources and Search Strategy

**Figure 1:**
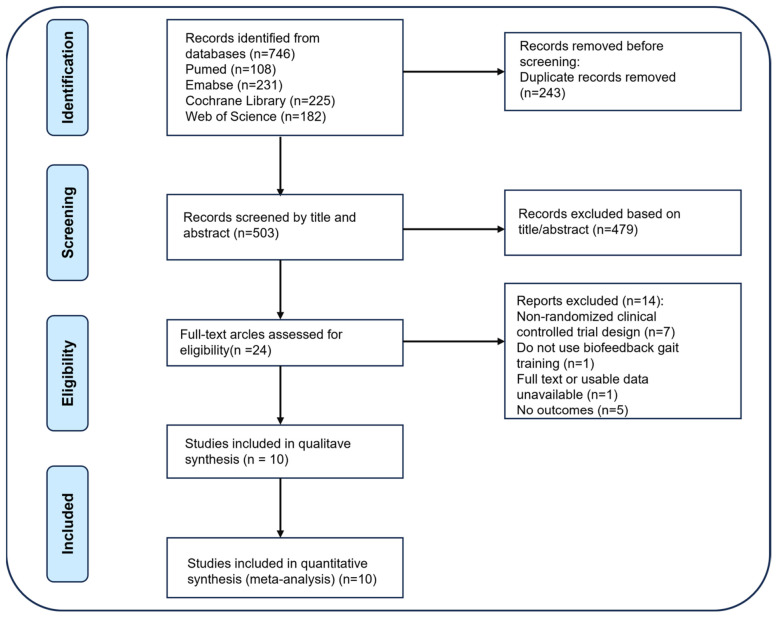
PRISMA flow diagram of literature screening.

Relevant studies were identified by searching PubMed, Embase, Web of Science and the Cochrane Library from inception to 7 April 2026. No language or publication-year restrictions were applied. Consistent with the PROSPERO registration, only published studies were sought, and no formal grey-literature or trial-registry search was performed to identify unpublished studies. Trial protocols or trial registration records were checked when available for included studies to assist the assessment of selective outcome reporting. The search terms for each database are provided in the Supplementary Table S1. Screening was performed by two independent reviewers. Figure 1 illustrates the search strategy based on the PRISMA guidelines [12].

### 2.2. Selection Criteria

Trials matching the following criteria were enrolled: (1) Randomized controlled trials (RCTs); (2) participants were stroke survivors with a clinically confirmed diagnosis of stroke and documented gait impairment; (3) the experimental intervention involved gait biofeedback training; (4) the comparator consisted of conventional rehabilitation, usual care, or comparable gait training without biofeedback; (5) at least five participants were included in each study arm, as a pragmatic minimum threshold to avoid extremely small treatment arms that may produce unstable estimates for continuous outcomes [13]; and (6) at least one spatiotemporal gait outcome was reported. The predefined primary spatiotemporal outcomes were gait velocity, step length, stride length, cadence, and stance time.

Studies were excluded if they met any of the following criteria: (1) non-randomized studies, observational studies, reviews, conference abstracts, protocols; (2) studies not involving stroke survivors or not reporting gait impairment; (3) studies did not include gait-related biofeedback training; (4) fewer than five participants in either study arm; (5) studies that did not measure any of the predefined spatiotemporal gait outcomes; and (6) Studies with conditions that could prevent safe participation in biofeedback gait training, such as severe osteoporosis.

### 2.3. Data Extraction and Quality Assessment

Two independent reviewers (KX. D and YQ. Y) screened the titles and abstracts of records identified from the databases. After duplicates were removed, the full texts of potentially eligible studies were assessed according to the predefined inclusion and exclusion criteria. Disagreements at either the title/abstract screening stage or the full-text screening stage were first resolved through discussion. If consensus could not be reached, a third reviewer (YJ. Y) arbitrated. Data extraction was also independently duplicated by two reviewers (KX. D and YQ. Y) using a standardized extraction form. Extracted information included country, participant characteristics, sex distribution, age, intervention details, training dosage, number of participants recruited and analyzed, and outcome measures. Where reported, potential patient-level factors related to treatment response were also recorded, including stroke chronicity, lesion side, baseline walking ability, cognitive status, sensory deficits, neglect, balance impairment, and assistive device use. Because these variables were inconsistently reported across studies, they were summarized descriptively. For incomplete or unclear numerical data, the full text, tables, figures, and Supplementary Materials were rechecked. No statistical imputation was performed. Studies with unavailable essential data were excluded from the relevant outcome-specific meta-analysis. When available, registration records were compared with published outcomes to assess selective reporting. The primary outcomes were spatiotemporal gait parameters, including gait velocity, step length, stride length, cadence, and stance time.

**Figure 2:**
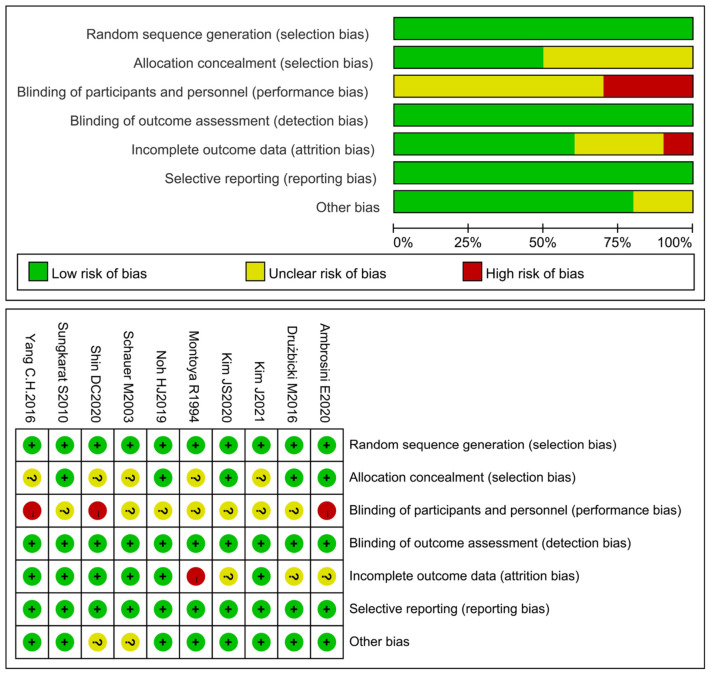
Cochrane Risk of Bias tool-1 (RoB-1) summary for the included studies [14,15,16,17,18,19,20,21,22,23]. Green, yellow, and red indicate low, unclear, and high risk of bias, respectively.

The Cochrane Risk of Bias tool (RoB-1) was used to evaluate risk of bias, in line with the Cochrane Handbook for Systematic Reviews of Interventions. RoB-1 evaluates seven domains, including random sequence generation, allocation concealment, blinding of participants and personnel, blinding of outcome assessors, incomplete outcome data, selective outcome reporting, and other sources of bias. Each domain was judged as having a low, high, or unclear risk of bias (Figure 2). Methodological quality was also assessed using the Physiotherapy Evidence Database (PEDro) scale. The PEDro scale contains 11 items, of which 10 are scored, with total scores ranging from 0 to 10. Higher scores indicate better methodological quality (9–10 excellent, 6–8 good, 4–5 fair, and <4 poor).

The certainty of evidence for each primary outcome was evaluated using the GRADE framework (Grading of Recommendations Assessment, Development and Evaluation) [24]. Two reviewers (KX. D and YQ. Y) independently assessed the evidence across five domains: risk of bias, inconsistency, indirectness, imprecision, and publication bias. Evidence certainty was classified as high, moderate, low, or very low. Disagreements were resolved through discussion.

### 2.4. Intervention Classification

Given the clinical and technical heterogeneity of the included interventions, we additionally classified each gait biofeedback intervention according to four prespecified dimensions: feedback modality, feedback target, delivery context, and measured outcomes. Feedback modality referred to the main sensory channel or technological form through which feedback was delivered, such as visual feedback, auditory feedback, musical motor feedback, or multimodal feedback. Feedback target referred to the main gait-related variable addressed by the intervention, such as step length, gait cadence, or gait symmetry. Delivery context referred to the training environment or task, including treadmill walking, overground walking, balance-platform training, or cycling-associated training.

Because the number of studies within each intervention category was small, formal subgroup analysis or meta-regression was not performed. Instead, a structured narrative synthesis was conducted to describe whether the measured outcomes were aligned with the feedback target and to explore potential sources of clinical heterogeneity across studies.

### 2.5. Statistical Analysis

All statistical procedures were performed with Review Manager 5.4 and Stata 18. All data were independently verified by two reviewers (KX. D and YQ. Y) for accuracy, and outcome data were examined for consistency in outcome definitions and measurement units. When the measurement methods and units were consistent across studies, MD was used as the effect size. Before pooling, outcome data were harmonized to common units. Gait velocity was expressed in centimeters per second (cm/s). Values reported in meters per second (m/s) were multiplied by 100, and values reported in meters per minute (m/min) were converted to cm/s. Step length and stride length were expressed in cm. Values reported in meters were multiplied by 100. Cadence was expressed in steps/min. Stance phase was pooled in seconds (s) when reported as absolute time. Percentage-based gait-cycle data were not combined with second-based data unless direct conversion was possible. Therefore, MD was used because the pooled outcomes were expressed in the same physical units and were clinically interpretable. All effect estimates were calculated with 95% CIs. Considering the expected clinical and methodological heterogeneity among rehabilitation trials, random-effects models were used as the primary analytical approach for all outcomes. Between-study heterogeneity was assessed using I^2^ and τ^2^ statistics [25]. Where possible, 95% PI were also calculated to estimate the range of effects that might be expected in future comparable clinical settings. Sensitivity analysis was performed when a single study had a disproportionate influence on the pooled estimate. Reporting bias was not formally assessed because fewer than 10 studies were available for each synthesis [26]. In addition, because only a small number of studies were available for each outcome, no formal subgroup analysis or meta-regression was performed. Instead, potential sources of heterogeneity were explored descriptively by comparing feedback modality, intervention dose, and outcome definitions across studies.

## 3. Results

### 3.1. Study Selection and Methodological Quality Assessment

A total of 746 records were retrieved from the initial searches of databases. After duplicate removal, 503 records remained for screening. Following full-text assessment, 10 RCTs were included in the qualitative synthesis and quantitative analysis [14,15,16,17,18,19,20,21,22,23]. Figure 1 presents the study selection process.

Methodological quality assessed using the PEDro scale is shown in Supplementary Table S2. PEDro scores ranged from 4 to 8, indicating overall fair-to-good methodological quality. Most studies satisfied the criteria for random allocation, baseline comparability, between-group statistical comparisons, and reporting of point estimates and variability. In contrast, blinding of participants and therapists was rarely achieved, which is common in rehabilitation trials involving training interventions.

According to the RoB-1 assessment, several risk-of-bias concerns were identified across the included studies (Figure 2). Random sequence generation, blinding of outcome assessment, and selective reporting were judged as low risk in all included studies. However, allocation concealment was rated as low risk in half of the studies, whereas the remaining studies were judged as unclear risk. Performance bias was the main methodological concern, as three studies [18,20,22] were rated as high risk, while the remaining studies were judged as unclear risk in this domain. For incomplete outcome data, most studies were assessed as low risk, although one study [14] was rated as high risk and three studies were judged as unclear risk. Other bias was generally low, with only two studies rated as unclear risk. Overall, four studies had at least one domain rated as high risk of bias, suggesting that the main methodological limitations were related to blinding procedures, allocation concealment, and, to a lesser extent, incomplete outcome data.

The GRADE assessment indicated very low to moderate certainty of evidence for the primary outcomes. Evidence certainty was moderate for gait velocity, low for step length and stride length, and very low for stance time. The summary of findings and detailed GRADE evidence profile for all outcomes are presented in Supplementary Table S3.

### 3.2. Study Characteristics

The key characteristics of the included studies are presented in Table 1 and Table 2. A total of 304 participants were included across the 10 RCTs. Among the 257 participants from eight studies that reported sex distribution, 150 (58.4%) were male. The studies were conducted in six countries across Europe and Asia: France, Germany, Thailand, Poland, Italy, and South Korea. The mean age of participants generally ranged from approximately 52 to 70 years. Patient-level characteristics that may influence responsiveness to gait biofeedback were incompletely reported across the included trials. Some studies described stroke stage or time since stroke, but information on baseline walking ability, balance, cognitive capacity, sensory deficits and neglect, and assistive device use was inconsistently available. Therefore, these factors could not be examined quantitatively as effect modifiers. The available information is summarized descriptively in Table 1.

The experimental interventions involved gait-related biofeedback training, either alone or combined with conventional rehabilitation. However, the specific protocols varied across studies in terms of feedback modality, feedback target, delivery context, and training dose. Visual feedback was delivered during treadmill training, overground walking, balance-platform training, or multimodal rehabilitation. Auditory feedback was mainly used to guide gait rhythm, step timing, or cadence. Whereas insole- or pressure-based systems provided feedback on weight bearing, foot contact, or walking performance.

The feedback targets also differed among studies. Some interventions directly targeted spatiotemporal gait parameters, such as step length and cadence, whereas others primarily addressed balance, trunk control, or limb motor control, with gait parameters measured as functional outcomes. Intervention dose is reported in Table 2, and the relationship among feedback modality, feedback target, delivery context, and outcomes is summarized in Table 3.

Comparator conditions also varied. Several studies [14,16,17,18] compared biofeedback-assisted training with similar walking or treadmill training without biofeedback, with generally comparable training time and task content. Other studies used conventional rehabilitation, general gait training, self-controlled walking, or multimodal training as comparators [15,19,20,21,22,23]. Overall, the comparator structure ranged from closely matched biofeedback-removed designs to broader comparisons with usual care or additional rehabilitation. These clinical and methodological differences should be considered when interpreting the pooled findings.

### 3.3. Meta-Analysis

**Figure 3:**
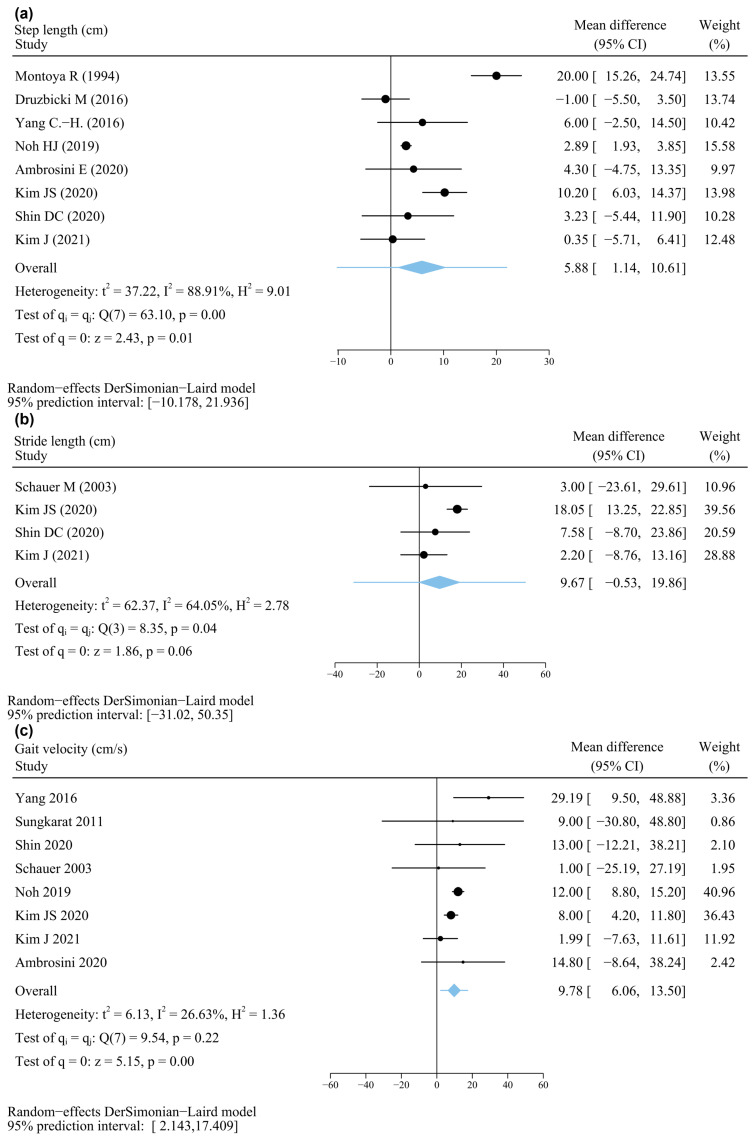

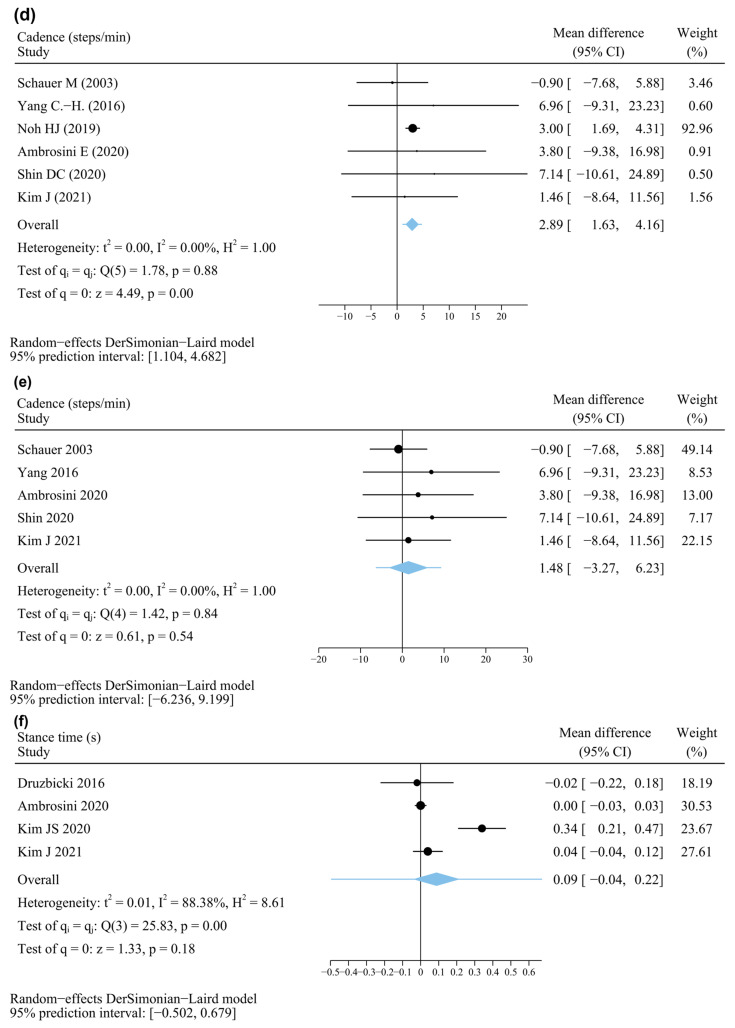
Forest plot of the effects of biofeedback gait training on (**a**) step length (cm) [14,15,18,19,20,21,22,23], (**b**) stride length (cm) [15,21,22,23], (**c**) gait velocity (cm/s) [15,16,18,19,20,21,22,23], (**d**) gait cadence (steps/min) [15,18,19,20,22,23], (**e**) sensitivity analysis for gait cadence after excluding the study by Noh et al. [19], and (**f**) stance time (s) [17,20,21,23]. The blue diamond represents the pooled MD and its 95% CI, and the blue horizontal line represents the 95% prediction interval. MD were based on post-intervention scores.

Eight studies [14,17,18,19,20,21,22,23] involving 210 participants contributed data to the meta-analysis of step length (Figure 3a). The random-effects model showed that gait biofeedback training may improve step length compared with control interventions (MD = 5.88 cm, 95% CI 1.14 to 10.61, *p* = 0.01). However, between-study heterogeneity was substantial (τ^2^ = 37.22, I^2^ = 88.91%), and the 95% PI was wide and crossed the null value (−10.18 to 21.94). This suggests that although the average effect favored biofeedback training, the effect may differ considerably across future settings and should be interpreted with caution.

Four studies [15,21,22,23] involving 116 participants contributed data to the meta-analysis of stride length (Figure 3b). The pooled analysis favored gait biofeedback training over control interventions, but the between-group difference was not statistically significant (MD = 9.67 cm, 95% CI −0.53 to 19.86, *p* = 0.06). Moderate-to-substantial heterogeneity was observed (τ^2^ = 62.37, I^2^ = 64.05%), and the 95% prediction interval was broad (−31.02 to 50.35). Therefore, the current evidence does not support a significant effect of gait biofeedback training on stride length.

Eight studies [15,16,18,19,20,21,22,23] involving 229 participants were included in the meta-analysis of gait velocity (Figure 3c). The random-effects model showed that gait biofeedback training was associated with a significantly greater gait velocity than control interventions (MD = 9.78 cm/s, 95% CI 6.06 to 13.50, *p* < 0.001). Heterogeneity was relatively low (τ^2^ = 6.13, I^2^ = 26.63%), and the 95% PI remained above zero (2.14 to 17.41). These findings suggest that gait biofeedback training may improve gait velocity across comparable rehabilitation settings, although the clinical importance of this effect should be interpreted in relation to established MCID thresholds.

Six studies [15,18,19,20,22,23] involving 170 participants were included in the meta-analysis of gait cadence (Figure 3d). The random-effects analysis showed that gait biofeedback training was associated with a significantly greater gait cadence than control interventions (MD = 2.89 steps/min, 95% CI 1.63 to 4.16, *p* < 0.00001). Statistical heterogeneity was not detected (τ^2^ = 0.00, I^2^ = 0.00%), and the 95% PI was 1.10 to 4.68. However, one study contributed a disproportionately large statistical weight to the pooled estimate. Sensitivity analysis excluding this study showed that the pooled effect was no longer statistically significant (MD = 1.48 steps/min, 95% CI −3.27 to 6.23, *p* = 0.54; I^2^ = 0%) (Figure 3e). These findings indicate that the apparent effect of gait biofeedback training on gait cadence was not robust and should therefore be interpreted with caution.

Four studies [16,20,21,23] involving 126 participants reporting stance time were included in the meta-analysis (Figure 3f). The pooled analysis showed no significant difference in stance time between gait biofeedback training and control interventions (MD = 0.09 s, 95% CI −0.04 to 0.22, *p* = 0.18). Heterogeneity was substantial (τ^2^ = 0.01, I^2^ = 88.38%), and the 95% prediction interval was wide (−0.50 to 0.68). This suggests that the effect of gait biofeedback training on stance time is uncertain and may vary markedly between studies.

Reporting bias was not formally assessed for any synthesis because fewer than 10 studies were available for each outcome.

## 4. Discussion

The present meta-analysis, using random-effects models for all outcomes, suggests that gait biofeedback training may improve selected spatiotemporal gait parameters in stroke survivors. This finding is generally consistent with previous pooled evidence suggesting beneficial effects of wearable real-time biofeedback on gait velocity after stroke and of rhythmic auditory stimulation on gait velocity and step length [27,28]. However, the strength of evidence differed across outcomes. For gait velocity, the pooled effect was statistically significant, heterogeneity was relatively low, and the PI remained above zero. For step length, although the average effect favored biofeedback training, the PI was wide and crossed the null value, indicating that the effect may not be reproduced in all future clinical settings. No significant pooled effects were found for stride length or stance time, and the apparent effect on cadence was not robust in the sensitivity analysis. Overall, the current evidence supports a cautious interpretation: gait biofeedback training may improve gait velocity, whereas the effects on other spatiotemporal gait parameters remain tentative, unstable, or uncertain.

The observed between-group difference in gait velocity may have potential clinical relevance. Gait velocity is one of the most widely used indicators of walking capacity after stroke and is closely related to functional ambulation and community participation. In the present meta-analysis, the pooled improvement in gait velocity was 9.78 cm/s, equivalent to 0.0978 m/s. This magnitude is close to the 0.10 m/s threshold that has been suggested as a substantial meaningful change in physical performance [29]. Therefore, the gait velocity finding may be clinically promising, but it should not be interpreted as definitive evidence that an established stroke-specific minimal clinically important difference (MCID) was achieved. Step length is also an important spatiotemporal parameter that reflects limb advancement, gait symmetry, and dynamic stability during walking. However, because no universally accepted MCID for step length after stroke is available, the clinical interpretation should remain cautious. A plausible explanation is that gait biofeedback provides immediate, task-specific information about walking performance and allows repeated error correction during practice [8]. Such external feedback may facilitate motor relearning by helping patients recognize and adjust abnormal gait patterns in real time [30,31].

The absence of significant pooled effects for stride length and stance time may be explained by several factors. First, fewer studies contributed to these analyses, which reduced statistical power and widened the CIs. Second, these outcomes may be less directly responsive to short-term feedback-based interventions than gait velocity or step length, particularly when training targets differed across studies. Third, stance time was reported inconsistently, with some studies using absolute time and others reporting a percentage of the gait cycle. Although stance time data were synthesized using consistent units where possible, this reduced the number of studies eligible for pooling. In addition, substantial heterogeneity was observed for step length and stance time, suggesting notable between-study variability. This heterogeneity may partly be explained by differences in participant characteristics, particularly the stage of stroke recovery. The studies contributing to these outcomes included both subacute participants within 6 months after stroke and chronic stroke survivors more than 6 months after stroke. In subacute participants, changes in step length or stance time may partly reflect spontaneous neurological recovery, early functional adaptation, and greater responsiveness to rehabilitation. By contrast, chronic stroke survivors may have more established compensatory gait patterns, and their responses may depend more on the feedback target, training dose, and baseline gait impairment. Therefore, pooling studies across different recovery stages may increase between-study variability and limit the direct applicability of the average effect to a specific clinical population.

The findings for gait cadence should also be interpreted cautiously. Although the primary meta-analysis suggested a significant benefit, the sensitivity analysis showed that this effect was not stable after removal of the most influential study. This suggests that the pooled estimate for cadence was strongly affected by a single trial. Clinically, cadence may also be more susceptible to compensatory changes than other gait parameters. An increase in cadence does not necessarily indicate a better overall gait pattern if it is not accompanied by corresponding improvements in step length, symmetry, or temporal stability.

Intervention heterogeneity was another important consideration. Given the differences in feedback modality, feedback target, training dose, and delivery context, a random-effects model was considered appropriate. Previous reviews have similarly suggested that the effects of post-stroke gait biofeedback may depend on intervention design and participant characteristics [8,32]. These differences suggest that the pooled estimates should be interpreted as average effects across clinically diverse biofeedback interventions. Some interventions directly targeted gait-related parameters, whereas others primarily focused on balance or trunk control. The mismatch between feedback targets and outcomes may partly explain the heterogeneity observed for step length and stance time, as well as the limited robustness of the cadence finding.

In addition to intervention heterogeneity, comparator imbalance should also be considered when interpreting the pooled effects. Control conditions differed in therapy time, walking practice, and intervention intensity. Although some trials more clearly isolated the biofeedback component, others compared biofeedback-based programs with usual care or additional multimodal training. Therefore, improvements in gait velocity may partly reflect nonspecific training effects, such as task repetition, therapist attention, or motivation, rather than the effect of biofeedback alone.

The PI provides further clinical information beyond the average pooled effects. For gait velocity, the PI remained above zero, suggesting that the beneficial effect may be relatively stable across comparable rehabilitation settings. In contrast, the PIs for step length, stride length, and stance time were wide and crossed the null value, indicating greater uncertainty and possible variation in future studies. Therefore, future trials should clearly report the active feedback component, intended motor target, delivery context, and training dose, and should select outcomes that are directly aligned with the feedback target.

Compared with previous reviews, the present study provides a more focused evaluation of spatiotemporal gait outcomes in stroke survivors. Earlier work by Spencer et al. was primarily a narrative review and highlighted the heterogeneity of feedback approaches [8]. Other recent reviews focused on more specific intervention types, such as haptic feedback, Wii Fit-based biofeedback, wearable real-time biofeedback devices, or rhythmic auditory stimulation, and often included broader outcomes such as balance, functional mobility, or activities of daily living [10,11,27,28]. Therefore, the effects on individual gait parameters remained less clearly defined. In contrast, the present review included only RCTs of gait biofeedback training in stroke survivors and synthesized key spatiotemporal gait parameters, including gait velocity, step length, stride length, cadence, and stance time. Analytically, we used random-effects models for all outcomes and calculated PIs where possible. This approach allowed us to distinguish relatively stable evidence for gait velocity from more tentative or unstable findings for other gait parameters.

Although gait asymmetry is a hallmark of post-stroke walking impairment and an important target of gait rehabilitation, symmetry-related outcomes were not included in the quantitative synthesis. This decision was based on methodological considerations. Across the included studies, symmetry-related outcomes were reported using different definitions and calculation methods, including gait symmetry, gait asymmetry, step length asymmetry, and side-specific spatiotemporal parameters. These outcomes were also expressed using different metrics, such as ratios, percentage differences, absolute differences, or separate paretic and non-paretic limb values. In addition, the direction of interpretation was not always consistent, because higher values indicated better symmetry in some measures but greater asymmetry in others. Therefore, pooling these data using a single MD would have risked combining clinically and statistically non-equivalent outcomes. To avoid misleading estimates, symmetry-related outcomes were not meta-analyzed in the present review.

This review has several strengths. Only RCTs were included, both RoB-1 and PEDro assessments were applied, and outcome-specific evidence was synthesized rather than combining broad and heterogeneous measures of walking performance into a single conclusion. Sensitivity analysis for gait cadence also helped identify the limited robustness of that finding. However, several limitations should be acknowledged. The total number of included studies was small, and sample sizes were generally modest. Some analyses were based on only four studies, reducing precision. Considerable clinical and methodological heterogeneity was present across interventions and outcome reporting. In addition, the lack of participant and therapist blinding is difficult to avoid in rehabilitation trials; it remains an important source of performance bias. Participants receiving biofeedback may have been more motivated or may have increased their effort during training, while therapists may have provided more encouragement, attention, or co-interventions to the experimental group. These factors could bias the observed effects in favor of gait biofeedback training, particularly for outcomes such as gait velocity and step length that are influenced by effort and task engagement. Unclear allocation concealment and incomplete outcome data in some trials may also have introduced selection or attrition bias.

From a clinical perspective, gait biofeedback training may be considered a promising adjunct to conventional rehabilitation, particularly when gait velocity is the main treatment target after stroke [33]. However, the current evidence is not sufficient to support strong or broad clinical implementation. This implication should be qualified by the GRADE findings. The evidence for step length was less certain, and the evidence for cadence, stride length, and stance time was unstable or insufficient. Therefore, current findings should be viewed as hypothesis-supporting rather than definitive guidance for clinical implementation. Future trials should adopt more standardized intervention protocols, provide clearer reporting of feedback targets, training schedules, and intervention fidelity, and use a more consistent selection of gait outcomes. Larger, adequately powered RCTs are also needed to determine which patients are most likely to benefit, whether certain feedback modalities are superior, and whether improvements in spatiotemporal parameters translate into sustained gains in functional ambulation and community mobility.

## 5. Conclusions

This meta-analysis suggests that gait biofeedback training may improve gait velocity in stroke survivors. However, the evidence for other spatiotemporal gait parameters remains tentative, insufficient, or very uncertain, and the observed effects should be interpreted cautiously because of small sample sizes, methodological heterogeneity, and risk-of-bias concerns in the included trials. Therefore, further high-quality RCTs with larger samples and standardized intervention protocols are needed to confirm these findings and to determine the most effective feedback modes, training targets, and dosing strategies.

## Figures and Tables

**Table 1:** Baseline characteristics of participants in the included studies.

Study	Post-Stroke Phase (Time Since Stroke)	Paretic Side (L/R)	Step Length (cm)	Stride Length (cm)	Gait Velocity (cm/s)	Gait Cadence (Steps/min)	Stance Phase (s)	Cognition, Sensory Deficits, or Neglect	Balance	Assistive Device Use
Montoya R (1994) [14]	Subacute (<6 months)	NR	E: 55 ± 2.5 C: 38.5 ± 4.3	NR	NR	NR	NR	MMSE = 24	NR	NR
Schauer M (2003) [15]	Subacute (<2 months)	E: 7/4 C 5/7	NR	E: 84 ± 30 C: 96 ± 30	E: 64 ± 30 C: 77 ± 30	E: 45.5 ± 8.5 C: 46.5 ± 7.6	NR	No severe impairment	NR	No assistive device
Sungkarat S (2010) [16]	Subacute (<6 months)	E: 7/10 C: 7/11	NR	NR	E: 23.52 ± 10.8 C: 22.24 ± 9.1	NR	NR	Follow instructions	BBS: 36–38; TUG: 32 s	Gait aid/AFO
Drużbicki M (2016) [17]	Chronic (>6 months)	E: 6/9 C: 6/4	E: 24 ± 7 C: 25 ± 6	NR	NR	NR	E: 1.12 ± 0.29 C: 1.05 ± 0.29	MMSE ≥ 24	NR	Usual aids
Yang C.H. (2016) [18]	Chronic (>6 months)	E: 4/7 C: 5/6	E: 33.18 ± 7.34 C: 33.35 ± 8.82	NR	E: 41.15 ± 20.16 C: 42.92 ± 20.24	E: 71.53 ± 17.94 C: 73.99 ± 20.5	NR	MMSE > 24	TUG: 24 s	NR
Noh HJ (2019) [19]	Subacute (<6 months)	E: 6/6 C: 5/7	E: 28.94 ± 1.85 C: 28.57 ± 1.86	NR	E: 47 ± 5 C: 46 ± 4	E: 64.01 ± 0.93 C: 63.87 ± 1.61	NR	MMSE ≥ 24	BBS: 42–43	NR
Ambrosini E (2020) [20]	Subacute (<6 months)	E: 13/21 C: 19/15	E: 45.3 ± 15.3 C: 42.4 ± 9.5	NR	E: 65.2 ± 27.9 C: 57.7 ± 18.7	E: 90.4 ± 24.5 C: 91 ± 15.1	E: 0.43 ± 0.07 C: 0.44 ± 0.08	MMSE = 26	BBS: 20–24	Walker available
Kim JS (2020) [21]	Chronic (>6 months)	E: 4/8 C: 4/8	E: 26.32 ± 6.03 C: 27.07 ± 6.1	E: 51 ± 10.77 C: 52.15 ± 11.71	E: 22 ± 4 C: 22 ± 4	NR	E: 0.37 ± 0.05 C: 0.35 ± 0.06	MMSE > 25	NR	Most used walking aids in daily life
Shin DC (2020) [22]	Chronic (>6 months)	E: 5/7 C: 4/8	E: 38.5 ± 9.98 C: 38.27 ± 8.44	E: 77 ± 20.37 C: 75.19 ± 15.47	E: 58 ± 20 C: 57 ± 24	E: 88.16 ± 16.36 C: 86.76 ± 21.9	NR	MMSE > 24	NR	NR
Kim J (2021) [23]	Chronic (>6 months)	E: 11/12 C: 6/16	E: 33.27 ± 12.74 C: 34.08 ± 12.11	E: 62.26 ± 21.97 C: 64.25 ± 20.77	E: 29.65 ± 15.18 C: 30.72 ± 14.2	E: 55.46 ± 13.15 C: 56.21 ± 12.75	E: 0.39 ± 0.14 C: 0.4 ± 0.14	MMSE > 24	TUG: 32 s; BBS: 29	Orthoses retained during GAITRite tests

Abbreviations: E/C, Experimental/Control; AFO, ankle-foot orthosis; MMSE: Mini-Mental State Examination; BBS: Berg Balance Scale; TUG: Timed Up and Go Test; NR, not reported.

**Table 2:** Characteristics of the included studies on gait biofeedback training after stroke.

No.	Author (Year)	Country	Group	Participants (M/F; Age)	Intervention	Training Dose	Outcomes	Outcome Time Point
1	Montoya R (1994) [14]	France	E	5/4; 64 (58–69)	CR + BF	5 × 6-m walks; 10 × BF-assisted walks	Step length	Post
			C	3/2; 60 (55–64)	CR + walking without BF	5 × 6-m walks; 10 × walks		Post
2	Schauer M (2003) [15]	Germany	E	NR (11); 59 ± 12	MMF-assisted gait training	20 min/d, 5 days/week, 15 sessions	Gait velocity; stride length; cadence	Post
			C	NR (12); 61 ± 12	CR	20 min/d		Post
3	Sungkarat S (2010) [16]	Thailand	E	12/5; 52.12 ± 7.17	CR with I-ShoWS	15 sessions, 60 min/session, 5 days/week, 2 weeks	Gait velocity	Post
			C	12/6; 53.83 ± 11.18	CR	15 sessions, 60 min/session, 5 days/week, 2 weeks		Post
4	Drużbicki M (2016) [17]	Poland	E	9/6; 61.9 ± 11.4	TT with visual BF	10 sessions, 30 min/session, 5 days/week, 2 weeks	Stance time; step length	Post
			C	5/5; 59.8 ± 11.7	TT without visual BF	10 sessions, 30 min/session, 5 days/week, 2 weeks		Post
5	Yang C.H. (2016) [18]	Korea	E	9/2; 51.91 ± 13.30	TT with RAF + CR	TT: 30 min/session, 3 sessions/week for 4 weeks; CR: 30 min/session, 10 sessions/week, 4 weeks	Gait velocity; cadence; stride length; step length	Post
			C	9/2; 55.82 ± 13.58	TT without RAF + CR	TT: 30 min/session, 3 sessions/week, 4 weeks; CR: 30 min/session, 10 sessions/week, 4 weeks		Post
6	Noh HJ (2019) [19]	Korea	E	7/5; 52.08 ± 12.81	CR + Space Balance 3D training	30 min/session, 5 days/week, 4 weeks	Gait velocity; cadence; step length	Post
			C	6/6; 56.33 ± 5.54	CR	1 h/session, 5 days/week, 4 weeks		Post
7	Ambrosini E (2020) [20]	Italy	E	21/13; 73.7 ± 11.7	Visual BF training with FES-assisted training + UC	VBF: 20 min/session; UC: 70 min/session; 5 days/week	Gait velocity; cadence; step length; stance time	Post
			C	17/17; 74.9 ± 12.8	UC + CR	90 min/session; 5 days/week		Post
8	Kim JS (2020) [21]	Korea	E	4/8; 54.75 ± 5.14	Overground walking training with visual BF	3 sessions/week, 6 weeks	Step length; stride length; gait velocity; stance time	Post
			C	4/8; 55.00 ± 4.86	CR and therapist-guided neuromuscular facilitation	30 min/session, 3 sessions/week, 6 weeks		Post
9	Shin DC (2020) [22]	Korea	E	NR (12); 57.75 ± 14.03	Visual BF trunk control training + CR	5 sessions/week, 4 weeks	Stride length; step length; gait velocity; cadence	Post
			C	NR (12); 59.25 ± 9.75	CR + occupational therapy + FES	5 sessions/week, 4 weeks		Post
10	Kim J (2021) [23]	Korea	E	14/9; 61.78 ± 8.06	Auditory BF using a smart insole + CR	60 min/session, 2 sessions/week, >3-day interval	Gait velocity; cadence; stride length; step length	Post
			C	13/9; 64.23 ± 0.57	CR + occupational therapy	60 min/day, 5 days/week		Post

Abbreviations: E/C, Experimental/Control; CR: conventional rehabilitation; BF: biofeedback; UC: usual care; FES, functional electrical stimulation; TT, treadmill training; I-ShoWS, Intelligent Shoes for Walking Speed; M/F, male/female; MMF, mechanomyographic feedback; NR, not reported; RAF, rhythmic auditory feedback.

**Table 3:** Taxonomy of gait biofeedback interventions and measured outcomes in the included studies.

Study	Feedback Modality	Feedback Target	Delivery Context	Measured Outcomes
Montoya R (1994) [14]	VBF + ABF	Step length, gait asymmetry	OW	Step length
Sungkarat S (2010) [16]	AFB	Walking symmetry, symmetrical weight distribution	OW	Gait velocity, step length asymmetry, stance time
Yang C.H. (2016) [18]	AFB	Cadence, gait rhythm	TT	Gait velocity, cadence, stride length, step length, gait asymmetry
Kim J (2021) [23]	AFB	Gait symmetry, heel contact	Smart insole-based gait training	Gait velocity, cadence, stride length, step length, gait symmetry
Schauer M (2003) [15]	MFB	Gait rhythm, gait timing	Walking	Gait velocity, stride length, gait cadence
Drużbicki M (2016) [17]	VFB	Gait symmetry, step length	TT	Stance time, step length, gait symmetry
Noh HJ (2019) [19]	VFB	Balance, postural control	Space Balance 3D training	Gait velocity, cadence, step length, stance time
Kim JS (2020) [21]	VFB	Gait symmetry	OW	Step length, stride length, gait velocity, stance time
Shin DC (2020) [22]	VFB	Trunk control	Sitting trunk control training	Step length, stride length, gait velocity, cadence
Ambrosini E (2020) [20]	Multimodal VFB	Balance, cycling-associated motor control	FES-assisted cycling	Gait velocity, cadence, step length, stance time

Abbreviations: VFB: visual feedback; AFB: auditory biofeedback; MFB: Musical feedback; OW: Overground walking; TT: Treadmill training.

## Data Availability

All data generated or analyzed during this study are included in the article/Supplementary Materials.

## Supplementary Materials

The following supporting information can be downloaded at: https://www.mdpi.com/article/10.3390/brainsci16070717/s1, Table S1: Search strategies; Table S2: Methodological quality of the included studies—assessed with the 11-item PEDro scale; Table S3: GRADE assessment results of meta-analysis and quality of evidence; PRISMA_2020_checklist_all [12].

## Abbreviations

The following abbreviations are used in this manuscript:

FES	Functional electrical stimulation
I-ShoWS	Intelligent Shoes for Walking Speed
M/F	Male/female
MMF	Mechanomyographic feedback
NR	Not reported
RAF	Rhythmic auditory feedback
